# Crossing Fibers Detection with an Analytical High Order Tensor Decomposition

**DOI:** 10.1155/2014/476837

**Published:** 2014-08-27

**Authors:** T. Megherbi, M. Kachouane, F. Oulebsir-Boumghar, R. Deriche

**Affiliations:** ^1^USTHB, FEI, LRPE, ParIMéd, Algiers, Algeria; ^2^Equipe-Projet Athena, Inria Sophia Antipolis-Méditerranée, France

## Abstract

Diffusion magnetic resonance imaging (dMRI) is the only technique to probe *in vivo* and noninvasively the fiber structure of human brain white matter. Detecting the crossing of neuronal fibers remains an exciting challenge with an important impact in tractography. In this work, we tackle this challenging problem and propose an original and efficient technique to extract all crossing fibers from diffusion signals. To this end, we start by estimating, from the dMRI signal, the so-called Cartesian tensor fiber orientation distribution (CT-FOD) function, whose maxima correspond exactly to the orientations of the fibers. The fourth order symmetric positive definite tensor that represents the CT-FOD is then analytically decomposed via the application of a new theoretical approach and this decomposition is used to accurately extract all the fibers orientations. Our proposed high order tensor decomposition based approach is minimal and allows recovering the whole crossing fibers without any a priori information on the total number of fibers. Various experiments performed on noisy synthetic data, on phantom diffusion, data and on human brain data validate our approach and clearly demonstrate that it is efficient, robust to noise and performs favorably in terms of angular resolution and accuracy when compared to some classical and state-of-the-art approaches.

## 1. Introduction

The diffusion magnetic resonance imaging or dMRI [1] is a magnetic resonance imaging (MRI) modality which is particularly suited to study and characterize the white matter neuronal architecture of the brain* in vivo* and noninvasively. The diffusion tensor model (DTI) introduced by Basser et al. in 1994 [2] was the first technique used for the fiber bundles reconstruction. However, due to the assumption of single fiber bundle per voxel, this technique is ineffective in regions where the fiber bundles intersect. Therefore, the fiber bundles reconstructed by tractography algorithms based on DTI are unreliable. To overcome the limitations of the DTI model, new high angular resolution techniques (HARDI) have been proposed, such as the diffusion spectrum imaging (DSI), the Q-ball imaging (QBI) [3, 4], or the symmetric high order tensors (SHOT) [5]. These techniques allow estimating the diffusion orientation distribution function (ODF) whose maxima are aligned with the orientations of the underlying fibers. The DSI technique provides the real ODF by measuring the diffusion signal on a whole 3D Cartesian grid in the q-space. However, this method is impractical in clinical studies because it requires an important acquisition time due to the huge number of samples and it requires too a very high gradient magnitude. The QBI model approximates the diffusion ODF function directly from the raw HARDI diffusion signal acquired from a spherical sampling of the diffusion space [3, 4]. Although the QBI-ODF contains the angular information by having its maxima aligned on the orientations of the underlying fibers, it has a low angular resolution by failing to reconstruct correctly the fibers crossing with angles less than 63° [3, 6]. A sharper function called fiber ODF or FOD function can be calculated from the ODF by using the spherical deconvolution techniques [7]. The FOD function has also its maxima aligned on the underlying fiber orientations; the FOD allows a gain in the angular resolution up to 15°. Traditionally the ODF and FOD functions are described in spherical harmonics (SH) basis; the angular resolution of these functions directly depends on the order of the SH basis: an acute angular resolution requires a high order of the SH basis. However, in case of higher orders, these ODF and FOD functions are prone to negative lobes due to noise. The high order symmetric tensors or equivalently the homogeneous polynomials have been proposed to model and reconstruct the FOD function [8] called CT-FOD for Cartesian tensor-FOD, whose maxima correspond exactly to the orientations of the underlying fibers. By imposing efficiently the positivity constraint, the CT-FOD function appears as an alternative to the FOD described in the SH basis. Furthermore, thanks to its polynomial form, the maxima of the CT-FOD function can be easily located. Therefore, in our work we start by estimating the CT-FOD function to reconstruct the symmetric high order tensor from the diffusion weighted-MRI (DW-MRI) data. Due to the importance of tractography and its increasing interest in clinical practice, it is important to accurately extract the fiber orientations to perform a reliable and accurate tractography. An efficient and accurate approach to perform this crucial and necessary preprocessing step is to extract the fibers orientations as the CT-FOD maxima. Different methods for extracting maxima of high order tensors exist in the literature; in [9], Bloy and Verma proposed to determine the fiber directions using the concept of Z-eigenvalues introduced by Qi in 2005 [10]. This maxima localization method, the so-called traditional method, suffers from a low angular resolution and does not allow recovering crossing fibers at angles below 60° [11]. The high order tensor decomposition in rank-1 tensor has been proposed to the maxima extraction issue in [12], and the low-rank decomposition approximation method known as CANDCOMP/PARAFAC (CP) [13] was used. It was proved in previous works [11, 12] that the tensor decomposition approaches recover the crossing fibers with a better angular resolution than the traditional methods of maxima localization. However, the CP-decomposition approximation requires predefining the tensor rank, that is, knowing the number of fibers in a voxel, which is impossible apriority. Furthermore, PARAFAC uses the alternating least squares (ALS) algorithm, which is a nonlinear optimization algorithm whose convergence is not guaranteed and depends on the initialization. In this paper, we propose to find the orientations of the fiber bundles from diffusion signals using an analytical decomposition of symmetric high order tensor; for lightness and clarity of the paper we will use the abbreviation Adecomp-SHOT for analytical decomposition of symmetric high order tensor. This Adecomp-SHOT method was initially proposed by Brachat et al. in 2010 [14] but remains in the theoretical field. However, the Adecomp-SHOT seems a priori interesting for the fiber orientations issue because, unlike the suboptimal CP-decomposition approximation, the Adecomp-SHOT is an analytical one and not restricted to subgeneric ranks. Thus, rather than CP-decomposition, Adecomp-SHOT would provide a minimal decomposition; this aspect is particularly interesting in the fiber orientations search since it would give the whole underlying fiber orientations without any apriority. Therefore, in the following we propose an original and efficient Adecomp-SHOT based approach to extract the fiber orientations from diffusion weighted-MRI data and we prove through many validations tests the effectiveness of the proposed method. The Adecom-SHOT is based on the SHOT; therefore we propose to use the CT-FOD for reconstructing the SHOT from the DW-MRI signal, since the CT-FOD constitutes the state-of-the-art. We begin this paper by presenting first the CT-FOD algorithm, before explaining the CP-decomposition and providing a detailed version of the Adecomp-SHOT algorithm; in this section we will also present the results of the intrinsic study done on both of Adecomp-SHOT algorithm and CP-decomposition. Then we describe our Adecomp-SHOT based approach to the fiber orientations search. Finally, we finish by presenting our validations and results on synthetic, phantom, and* in vivo* human brain data and our conclusions.

## 2. Materials and Methods

### 2.1. Symmetric Fourth Order Tensor Coefficients from the Diffusion Data

The diffusion signal *S* = (*g*_*i*_, *b*_*i*_) corresponding to the acquisition parameters, *g*_*i*_, *b*_*i*_, is given by the convolution of the CT-FOD function *F*, modeled by a Cartesian and positive definite symmetric high order tensor of 3 dimensions, with a Watson function *Ω* [7, 8]:

(1) $$\begin{array}{c} S\left(g,b\right)=\Omega \left(g,b\right)⊗F\left(g,b\right) \end{array}$$

(2) $$\begin{array}{c} S\left(g_{i},b_{i}\right)=\int \Omega \left(g_{i},b_{i},v\right)F\left(v\right)dv \end{array}$$

*Ω*(*g*_*i*_, *b*_*i*_, *v*) = *e* ^−*b*_*i*_*D*(*g*_*i*_^*T*^*v*)^2^^, with *D* being the diffusivity coefficient calculated from a 2nd order tensor, that we estimate from a single fiber response having a high fractional anisotropy (FA > 0.8); *g*_*i*_ is the gradient of magnetic fields, *b*_*i*_ stands for the weights* b*-values, and *v* represents a set of unit vectors sampling the diffusion space.  Algorithm 1 describes the estimation of unique coefficients of the symmetric tensor FOD from the diffusion data.

In the following we are interested to decompose symmetric fourth order tensors of dimension 3 (*d* = 4 and *n* = 3).

### 2.2. Symmetric Tensor Decomposition

Symmetric high order tensors appear mostly as multivariate functions (more than two variables), and high order tensors decomposition allows deducing the geometric and invariance properties of a tensor. Therefore, the tensor decomposition raises interest in many practical domains, first in chemometrics [13] and psychometrics fields and then in electrical engineering and electronics [16], in particular for the antenna array processing [17], or else in telecommunication field [18]. Also, tensor decomposition appears very useful in data analysis and in the arithmetic complexity area [19]. Recently the interest in tensor decompositions has expanded to the neurosciences field; we cite among several applications the use of the symmetric tensor decomposition to the problem of extracting the fiber orientations of the white matter in dMRI. However, to date, in dMRI the decomposition problem is still solved with a low-rank approximation method known as CANDCOMP/PARAFAC (CP).

In the following two subsections, we first start by describing the classical CANDECOMP/PARAFAC (CP) method to decompose symmetric high order tensors and then present in detail the Adecomp-SHOT approach we propose to analytically decompose a symmetric tensor of any order and any dimension in a minimal sum of rank-1 terms.

#### 2.2.1. Numerical Method: CP Low-Rank Approximation

The tensor decomposition problem consists in writing a given tensor, in sum of outer product of vectors, that is, rank-1 tensor, and that with a minimal number of terms, the number of terms corresponding to the minimal tensor rank. Considering a symmetric tensor *T* of order *d* and dimension *n*, the minimal decomposition of this tensor should be in the following form:

(3) $$\begin{array}{c} T=\overset{R}{\underset{r=1}{\sum }}V_{r}^{1}\circ V_{r}^{2}\circ \cdots \circ V_{r}^{d} \end{array}$$

with *R*: the rank of *T*, “∘”: the outer product, and *V*_*r*_: the rank-1 tensors (vectors).

However, determining the rank of high order tensors (order >2) is a hard mathematical and NP-complet problem. Therefore, a low-rank numerical approximation of the decomposition (4) has been proposed in [13], where the authors approximate the tensor by another tensor whose rank is inferior to the minimal or generic tensor rank; this numerical decomposition method is known as CANDECOMP/PARAFAC (CP):

(4) $$\begin{array}{c} \hat{T}\approx \overset{k}{\underset{r=1}{\sum }}\lambda_{r}\left(v_{r}^{1}\circ v_{r}^{2}\circ \cdots \circ v_{r}^{d}\right) \end{array}$$

with ||*v*_*r*_|| = 1 and *λ*_*r*_: the weights of the rank-1 tensors *v*_*r*_.

*k* < *R* is the subgeneric rank of the tensor *T*, for symmetric tensors *v*_*r*_^1^ = *v*_*r*_^2^ = ⋯ = *v*_*r*_^*d*^. Thus, the CP-decomposition of the tensor *T* of order *d* and of an unknown minimal rank *R* is done by a nonlinear minimization in $\hat{T}$ of (5) for a given subgeneric rank *k*; the nonlinear minimization problem is solved using the alternating least squares algorithm (ALS):

(5) $$\begin{array}{c} \underset{\hat{T}}{\min}||\hat{T}-T||. \end{array}$$

*Inverse Problem*. To evaluate the intrinsic behavior of the CP method, we have simulated an inverse problem by generating fourth order symmetric tensors of rank-2 according to (3). These tensors are constructed from two crossing vectors with variable angles from 90° to 0° and weighted by the same weight *λ* = 0.5. The purpose is the evaluation of both the ability of the method to render the correct solutions and the angular resolution in such ideal case where data perfectly satisfy the decomposition model.

**Figure 1:**
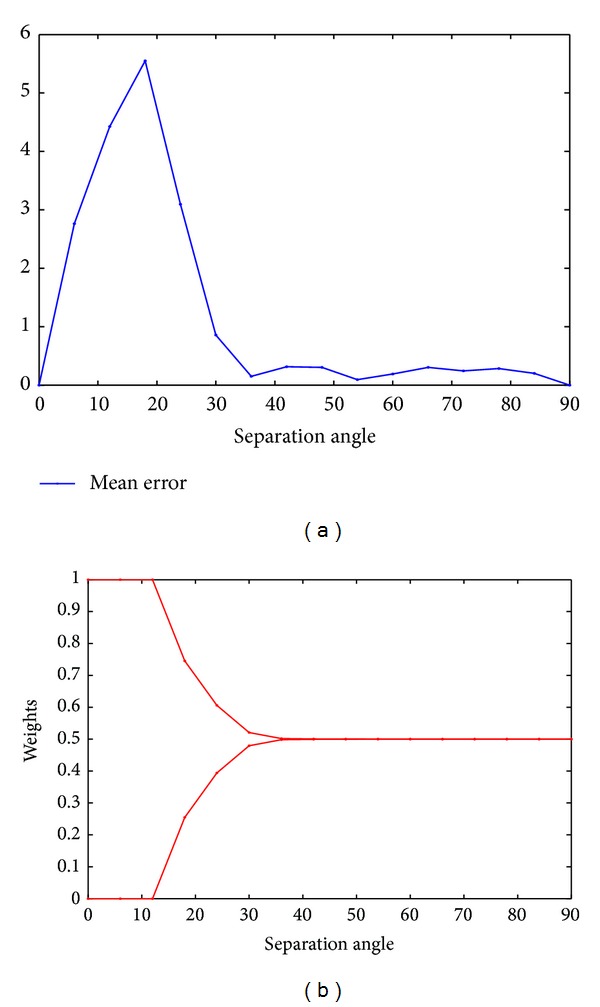
Rank-2 symmetric fourth order. (a) The mean error. (b) The weights *λ* of the rank-1 tensors. Horizontal axes (a-b): the separation angles from 90° to 0°.

The results of the intrinsic study are illustrated in Figure 1; Figure 1(a) represents the mean error between the simulated vectors or rank-1 tensors and the CP-decomposition solutions, and Figure 1(b) gives the rank-1 tensors weights found by the CP-decomposition, according to the separation angles.

From Figure 1(a) we notice that although the tensors to decompose are constructed as to satisfy the decomposition model described in (3), the CP-decomposition begins to give an incorrect decomposition when the solutions are separated with small angles (<30°); thus, Figure 1(b) shows that only one vector of the two simulated is detected with the weights *λ*_1_ = 1 and *λ*_2_ = 0 for crossing angles inferior to 10°. We conclude that the accuracy and the angular resolution of the CP-decomposition are intrinsically limited. Furthermore, the CP-decomposition has another important limit which is the requirement to predefine the rank of the decomposition.

The use of the CP-decomposition algorithm in dMRI to detect the fiber orientations was proposed in 2011 by Jiao et al. [12]. The authors considered an approximation of the decomposition with a low-rank value (rank = 2) in order to extract two crossing fiber orientations. The extracted fiber orientations correspond to the rank-1 vectors obtained from the decomposition. Although tensors decomposition is more efficient in terms of angular resolution and accuracy than the traditional maxima localization methods [11, 12], the CP-decomposition could not guarantee the recovering of the whole underlying fiber orientations since the minimal rank of the tensor is not a priori known. Moreover, the convergence of the ALS algorithm is not guaranteed and depends on the initialization.

In 2010, Brachat et al. proposed [14] to solve the symmetric high order tensor decomposition problem analytically; in the remainder of the paper we denote this method: Adecomp-SHOT. Rather than the CP numerical approach, the Adecomp-SHOT method gives a minimal decomposition without any apriority. However, to date the Adecomp-SHOT remains theoretical and not yet expanded to the practical issues or evaluated on physical phenomenons. Due to its ability to render a minimal decomposition, we naturally expect that the Adecomp-SHOT applied to the fiber orientations search in dMRI would be more interesting than the CP low-rank approximation method.

#### 2.2.2. Analytical Method: Adecomp-SHOT

The Adecomp-SHOT method initially proposed by the authors Brachat et al. [14] is a generalization of the Sylvesters theorem [14], initially introduced for binary cases and extended to larger dimensions. Thus, the Adecomp-SHOT algorithm is able to decompose a symmetric tensor of any order and any dimension, in a minimal sum of rank-1 terms.

Consider a symmetric tensor of order *d* and dimension *n* given in the following polynomial form:

(6) f(x0,x1,…,xn) =∑j0+j1+⋯+jn=dCj0,j1,…,jnx0j0x1j1…xnjn

with *f*(*x*_0_, *x*_1_,…, *x*_*n*_) ∈ *R*_*d*_ a homogenous polynomial of order *d* in *n* variables, and *C*_*j*_0_,*j*_1_,…,*j*_*n*__ the coefficients of the homogenous polynomial *f*.

An affine decomposition of *f* consists in writing *f* as a sum of *d*th powers of rank-1 linear forms [14] as follows:

(7) f(X)=∑i=1λiKi(X)d,f(X)=λ1K1(X)d+λ2K2(X)d+⋯+λrKr(X)d

with *λ*_*i*_ representing scalars weights, *K*_*i*_(*X*) rank-1 linear forms in *X* = [*x*_0_, *x*_1_,…, *x*_*n*_] with the coefficients (*k*_*i*,0_, *k*_*i*,1_,…, *k*_*i*,*n*_), and *r* the minimal rank of *f*, that is, of the tensor.

*K*_*i*_(*X*) is a rank-1 linear form of *n* dimensions [14]:

(8) $$\begin{array}{c} K_{i}\left(X\right)=k_{i,0}\cdot x_{0}+k_{i,1}\cdot x_{1}+\cdots +k_{i,n}\cdot x_{n} \end{array}$$

with *k*_*i*,0_ ≠ 0 for 1 ≤ *i* ≤ *r*.

An affine decomposition of *f* exists, if and only if an affine decomposition of $\underline{f^{*}}$ exists; thus, the decomposition of *f* is equivalent to decomposing $\underline{f^{*}}$; with f* being the linear form associated with *f* in the dual space *R*_d_*, the coefficients C* of f* are then calculated from the coefficients *C* of *f* [14] as follows:

(9) $$\begin{array}{c} C_{j_{0},j_{1},\ldots ,j_{n}}^{*}=\frac{j_{0}!j_{1}!\ldots j_{n}!}{d!}\times C_{j_{0},j_{1},\ldots ,j_{n}}. \end{array}$$

$\underline{f^{*}}$ is obtained by unhomogenizing f* according to the variable *x*_0_; this is done by dividing each monomial of the homogenous polynomial f* by an appropriate power of *x*_0_. Thus, $\underline{f^{*}}$ is a nonhomogenous polynomial of degree *d*.

The necessary and sufficient existence conditions of the decomposition are based on the rank conditions of the Hankel matrix and the commutation properties. The Hankel matrix is a *N* × *N* matrix with

(10) $$\begin{array}{c} N=\frac{\left(n+\left(d-1\right)\right)!}{d!\left(n-1\right)!}. \end{array}$$

*N* corresponds to the number of unique coefficients of a *d*-order symmetric tensor of *n* dimensions. The elements of the Hankel matrix are computed from the coefficients C* of f*; we note that the elements corresponding to monomials with total degree higher than the polynomial degree *d* are unknown. We provide an appendix (Appendix section) where we give an example of a step by step decomposition of a 3 dimensional 4th order tensor of rank-4, illustrating how the Adecomp-SHOT algorithm works and particularly we show in Step 2 of the Appendix section how the Hankel matrix is constructed from a homogenous polynomial.

To obtain the points *k*_*i*_ of (8), we calculate from the Hankel matrix the multiplication matrix *M*_*i*_ as described in Step  5 of Algorithm 2, with *i* = 1,…, *r*, for a given rank *r*; then we resolve the generalized eigenvalue problem and we deduce the weights *λ*_*i*_ by simply resolving a linear system. The readers can refer to the example given in the Appendix section for much more details. The critical part of Algorithm 2 is the step of extending the Hankel matrix, Step  6 of Algorithm 2, to verify the stability of the rank in case of higher ranks. Indeed, when the Hankel matrix is not totally defined, the extension requires finding the unknown parameters *h* of the Hankel matrix satisfying the commutation properties of the multiplication matrix *M*_*l*_ · *M*_*m*_ − *M*_*m*_ · *M*_*l*_ = *∅* with *l*, *m* = 1,…, *n*; this leads to solving a nonlinear equations system. Therefore, for higher ranks the uniqueness of the decomposition is not guaranteed.

**Figure 2:**
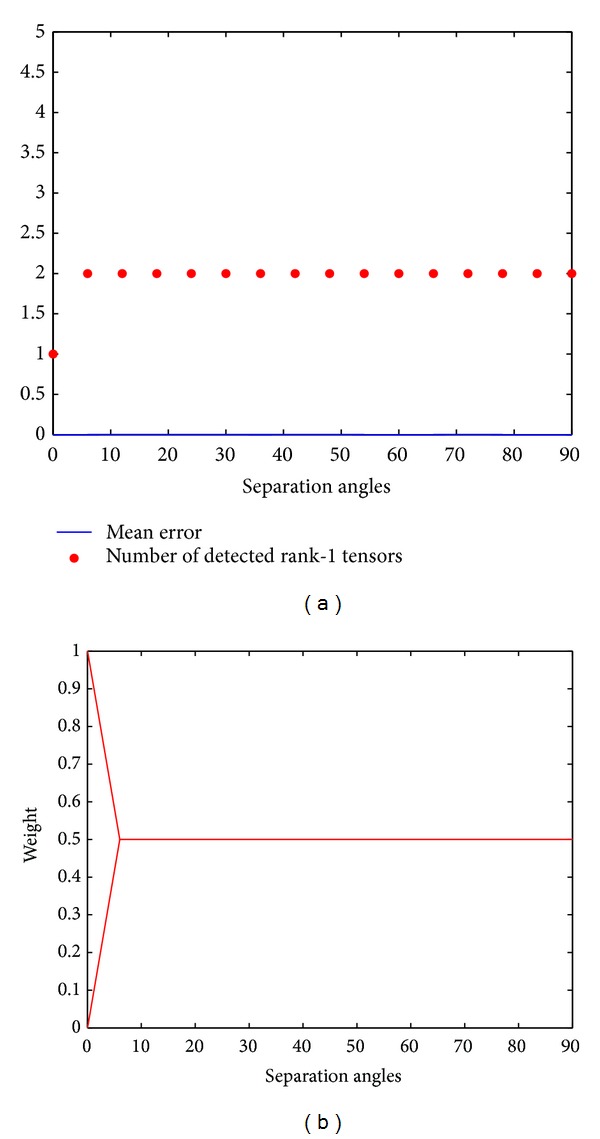
Rank-2 symmetric fourth order. (a) Blue: the mean error. (a) Red: the number of detected rank-1 tensors. (b) The weights *λ* of the rank-1 tensors. Horizontal axes (a-b): the separation angles from 90° to 0°.

*Inverse Problem.* Once again, we have simulated an inverse problem, this time for the Adecomp-SHOT algorithm; thus, rank-2 fourth order tensors were constructed from a sum of two rank-1 linear forms of order four, as described in (7). We have simulated the same configuration as in Section 2.2.1 by constructing the fourth order tensors from two crossing rank-1 linear forms at varying angles from 90° to 0° and weighted by the same weight *λ* = 0.5. Our results of the intrinsic study of the analytical decomposition method are thus given in Figure 2.

**Figure 3:**
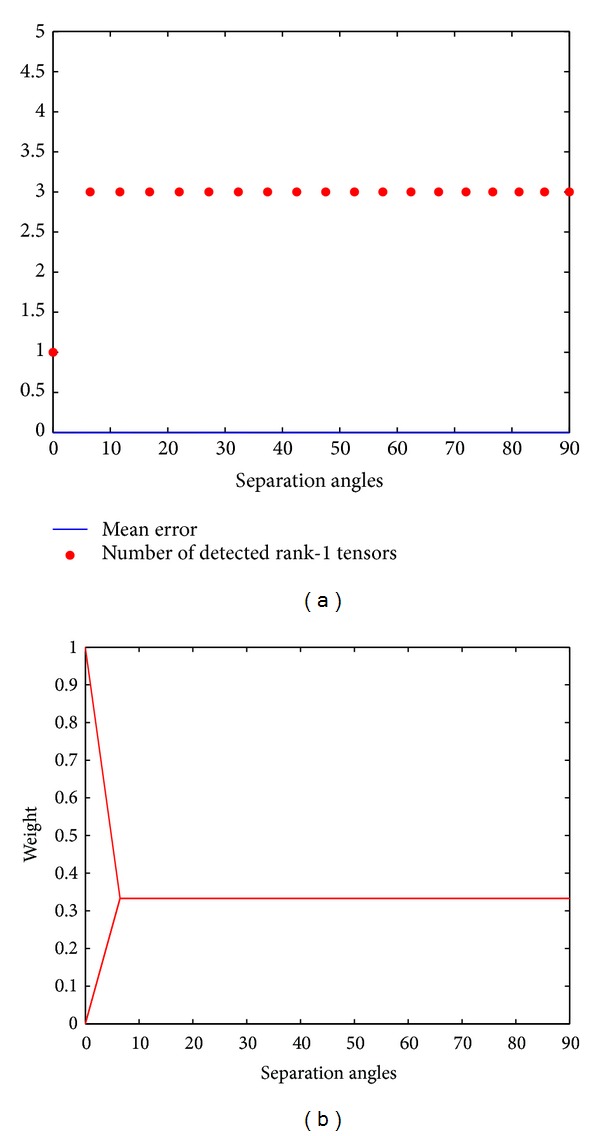
Rank-3 symmetric fourth order. (a) Blue: the mean error. (a) Red: the number of detected rank-1 tensors. (b) The weights *λ* of the rank-1 tensors. Horizontal axes (a-b): the separation angles from 90° to 0°.

Contrarily to the CP-decomposition, the analytical approach clearly renders the correct decomposition whatever the separation angles. As shown in Figure 2(a) the obtained mean error is zero, and the two rank-1 tensors are recovered with the correct weights *λ*_1_ = *λ*_2_ = 0.5 as represented in Figure 2(b). Furthermore, the analytical decomposition is minimal; that is, the rank of the tensor is automatically found without any assumption such that it is required for the CP-decomposition. To confirm the ability of the method to give always a minimal decomposition regardless of the rank of the tensor, further tests on higher rank tensor have been conducted; Figure 3 shows the results of decomposing a rank-3 symmetric fourth order tensor constructed from 3 crossing rank-1 tensors, according to (7), at angles decreasing from 90° to 0°. Figure 3 shows that the Adecomp-SHOT method gives once again a correct decomposition with a zero mean error whatever the separation angles between the 3 origin rank-1 tensors. Other tests on symmetric fourth order tensors of rank-4 and rank-5 have been conducted; the results of these experiences show that the Adecomp-SHOT method still gives the correct decomposition in case of rank-4 while for a rank-5 fourth order tensors the decomposition is found with insignificant angular error not exceeding 0.5°. However, by increasing the order of the tensor from 4 to 6 the error drops to zero.

### 2.3. Fiber Directions from Diffusion Data Using the Analytical Decomposition of Fourth Order Tensor

In this section we propose to extract the fibers orientations from the dMRI signal by decomposing analytically the three dimensional fourth order CT-FOD using the Adecomp-SHOT. The coefficients of the fourth order FOD are estimated from the dMRI data as described in Section 2.1. Thus, we are interested to decompose the Cartesian FOD tensor in sum of powers of rank-1 linear forms:

(11) $$\begin{array}{c} f\left(g\right)=\overset{r}{\underset{i=1}{\sum }}\lambda_{i}K_{i}{\left(g\right)}^{4} \end{array}$$

with *r* minimal representing the tensor rank; *K*_*i*_ are rank-1 linear forms in 3 variables with the real normalized coefficients [*k*_*i*,0_, *k*_*i*,1_, *k*_*i*,2_], and *λ*_*i*_ are the real positive weights.

The normalized coefficients of *K*_*i*_ represent the Cartesian coordinates of the fibers orientations, weighted by the scalar *λ*_*i*_, and *r* represents the number of crossing fibers bundles in each voxel.

However, as described in Section 2.2.2 the affine decomposition of a homogenous polynomial of any order and any dimension is done assuming that all coefficients of the first Cartesian coordinate in the decomposition are nonzero; that is, *k*_*i*,0_ ≠ 0, for 1 ≤ *i* ≤ *r*, with *r* the tensor rank. This constraint implies that only fiber orientations whose first coordinate coefficient is nonzero can be detected. To avoid missing fiber orientations, we propose to introduce a coordinate changing in case where maxima are located in undetectable area. Also, not doing this coordinate transformation systematically and imposing a stopping criterion, we propose to make a first exhaustive search on the FOD by discretizing it on unit sphere and then localize roughly its maxima. A given FOD function *f*(*g*), with *g* the gradient of the magnetic field, can be discretized on units sphere as follows:

(12) $$\begin{array}{c} f\left(g\right)=\overset{N^{\prime }}{\underset{j=1}{\sum }}p_{j}f_{j}\left(v_{1}^{j},v_{2}^{j},v_{3}^{j}\right) \end{array}$$

with *N*′ the number of samples; *p*_*j*_ *f*_*j*_ is a rank-1 fourth order tensor constructed in the orientation (*v*_1_^*j*^, *v*_2_^*j*^, *v*_3_^*j*^) and represents the value of the FOD in this orientation, and the 3 dimensional unit vectors *v*_*j*_ are obtained by a uniform tessellation of unit sphere. Thus, the *p*_*j*_ are found by solving the linear system *B* = *WP*, with *W* representing a vector of *N*′ weights *p*_*j*_, *B* vectors of 15 unique coefficients of the fourth order tensor FOD and *P* a (15 × *N*′) matrix; *P* contains the 15 unique coefficients of the *N*′ rank-1 fourth order tensor constructed from unit vectors *v*. Once the FOD is discretized, we check the first coordinate of the FOD values weighted by *p*_*j*_ < (0.5 × *p*_max⁡_) corresponding to the FOD lobes, and we make a coordinate changing before decomposing the tensor if these coordinates are close to zero. Obviously, after decomposition, the inverse coordinate transformation is required to bring back the resulted rank-1 tensor to the origin coordinate system. In order to preserve the angles between two vectors and their lengths, the transformation matrix should be orthogonal. Therefore, to preserve the fiber orientations we use a rotation transformation; considering the initial coordinates or variables *X* = [*x*_0_, *x*_1_,…, *x*_*n*_], *X*′ = [*x*_0_′, *x*_1_′,…, *x*_*n*_′] is obtained from *X* by the following linear transformation:

(13) x0′=a11x0+a12x1+⋯+a1nxnx1′=a21x0+a22x1+⋯+a2nxn⋮xn′=an1x0+an2x1+⋯+annxnX′=M×X

As mentioned, the matrix *M* is an orthogonal matrix or a rotation matrix. This coordinate transformation can be done either on the homogenous polynomial as a change of variable using a rotation transformation or simply by rotating the fourth order tensor coefficients. This coordinate transformation insures recovering the entire crossing fibers whatever its locations in the Cartesian space.

The Adecomp-SHOT as initially described in [14] would provide a minimal decomposition with a minimal analytical rank, without any constraints on the weights *λ*_*i*_ or *K*_*i*_ values; in theory this is not a problem, but when we are interested in detecting the fibers orientations, negatives values for the weights or complexes coefficients do not correspond to any physical meaning. To overcome this limit, we propose ignoring the *K*_*i*_ with complexes coefficients found at Step  7 of Algorithm 2 and then solve the linear system (14) with only the linear forms *K*_*i*_ with real coefficients:

(14) $$\begin{array}{c} f\left(g\right)\approx \overset{r^{\prime }}{\underset{i=1}{\sum }}\lambda_{i}K_{i}{\left(g\right)}^{4} \end{array}$$

*r*′ ≤ *r* represents the tensor rank and *K*_*i*_ ∈ *R*_4_. This linear system can be written as a matrix equation *B* = *WA*, with *W* a vector of length *r*′ containing the fibers weights *λ*_*i*_ and *A* is a (*r*′ × 15) matrix containing the polynomials coefficients of the rank-1 linear forms *K*_*i*_(*g*)^4^ of order 4 and *B* is a vector of length 15 containing the coefficients of the fourth order fiber orientation distribution function *f*(*g*). To impose the positivity constraint on the weights *λ*_*i*_ values, we propose to solve the minimization problem 2.17 using the well-known Lawson and Hansons NNLS algorithm [15]:

(15) $$\begin{array}{c} \underset{w}{\min}{||B-WA||}^{2}. \end{array}$$

However, to verify that a set of *λ*_*i*_ > 0 such that *f*(*g*) ≈ ∑_*i*=1_^*r*′^ *λ*_*i*_ *K*_*i*_(*g*)^4^ exists we check the norm ||*B*−*WA*||^2^; if the residual norm is less than 1, we assert that it is sufficient to consider the resulted decomposition accurately and the *K*_*i*_ weighted by *λ*_*i*_ > 0 correspond to the maxima of *f*(*g*); else, we propose to relaunch the decomposition by doing a change of coordinates. This trick allows imposing the positivity constraint and increasing the accuracy of the decomposition.

Finally, to take into account the effect of the noise due to the diffusion model, we have introduced a heuristic cleaning that consists in removing all the fiber orientations weighted by *λ*_*i*_ ≤ 0.1*λ*_max⁡_ and merging fibers separated by angle *α* ≤ 15° [20].

## 3. Results and Discussion

**Figure 4:**
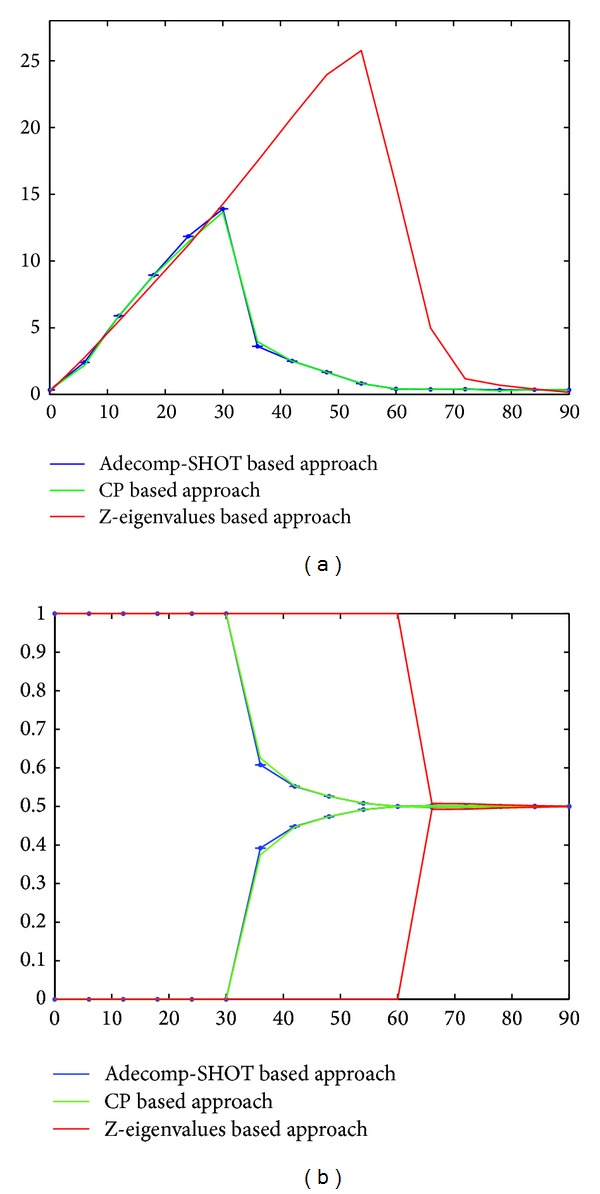
Free-noise synthetic diffusion data: (a) solid lines: mean error in degree; (a) black dot: the number of detected fibers by the Adecomp-SHOT based approach. (b) The weights *λ*. (a-b) Red: the Z-eigenvalues based approach; green: the CP-decomposition based approach; blue: the Adecomp-SHOT based approach. Horizontal axes (a-b) represent the separation angles from 90° to 0°.

**Figure 5:**
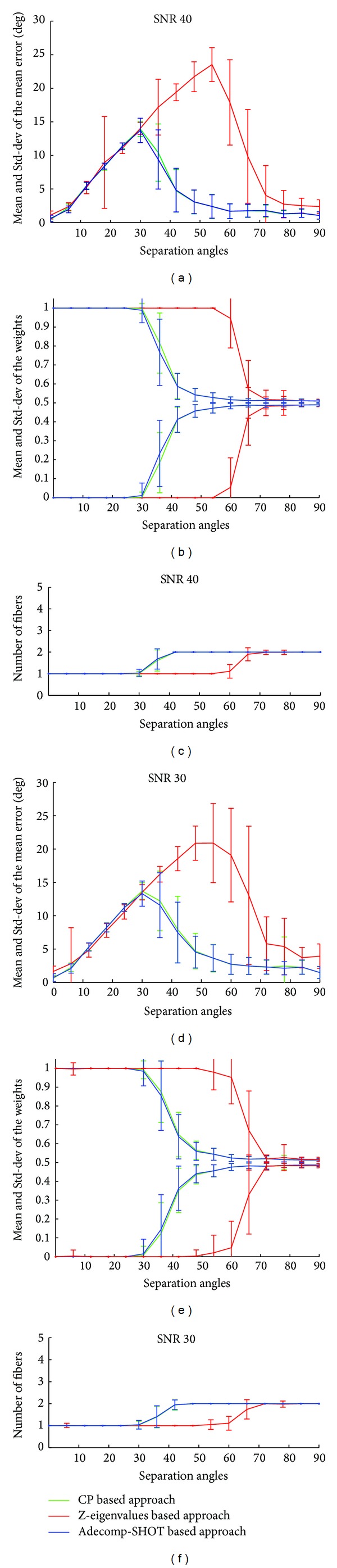
Noisy synthetic data with SNR 40 and 30: (a, b, and c) SNR 40 and (d, e, and f) SNR 30. (a, d) The mean and the standard deviation of the mean error; (b, e) the mean and the standard deviation of the fiber weights *λ*; (c, f) the mean and the standard deviation of the number of detected fibers. Horizontal axes (a–f) the separation angles from 90° to 0°.

**Figure 6:**
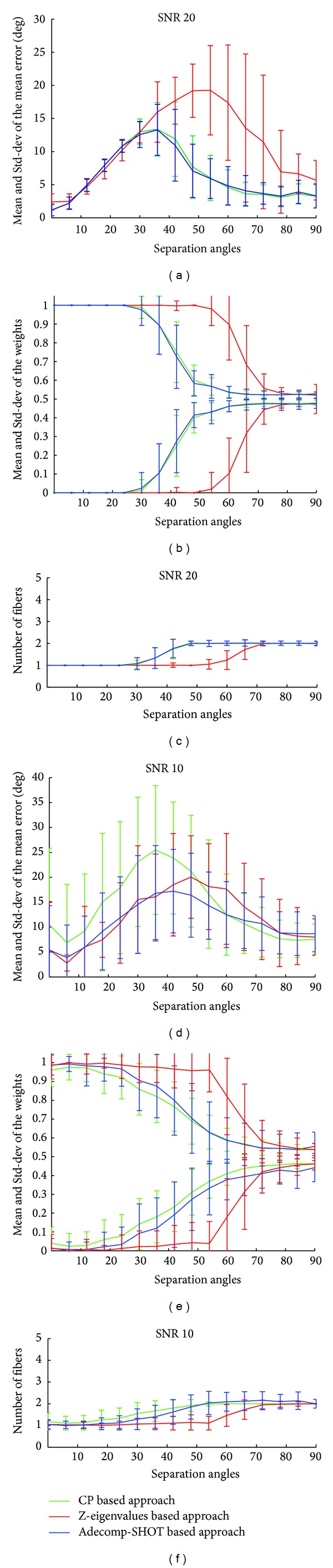
Noisy synthetic data with SNR 20 and 10: (a, b, and c) SNR 20 and (d, e, and f): SNR 10. (a, d) The mean and the standard deviation of the mean error; (b, e) the mean and the standard deviation of the fiber weights *λ*; (c, f) the mean and the standard deviation of the number of detected fibers. Horizontal axes (a–f) the separation angles from 90° to 0°.

**Figure 7:**
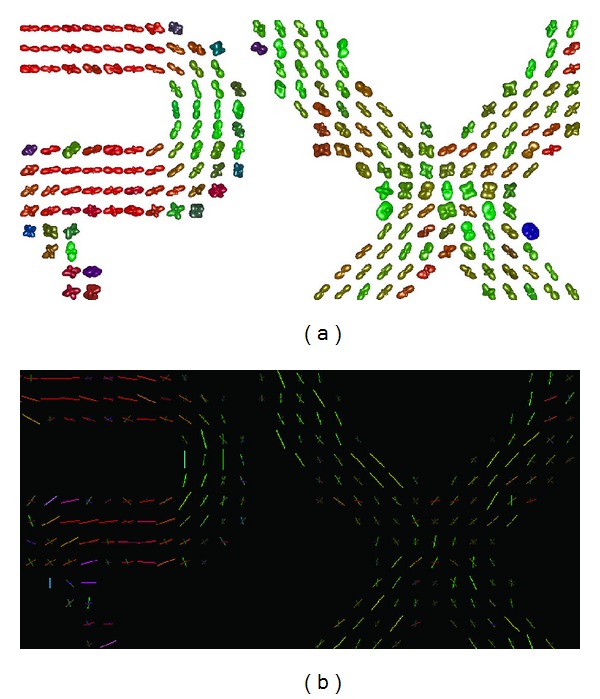
(a) Fourth order CT-FODs reconstructed from FiberCup data. (b) Fiber directions corresponding to the fourth order CT-FODs maxima extracted using the Adecomp-SHOT based approach.

**Figure 8:**
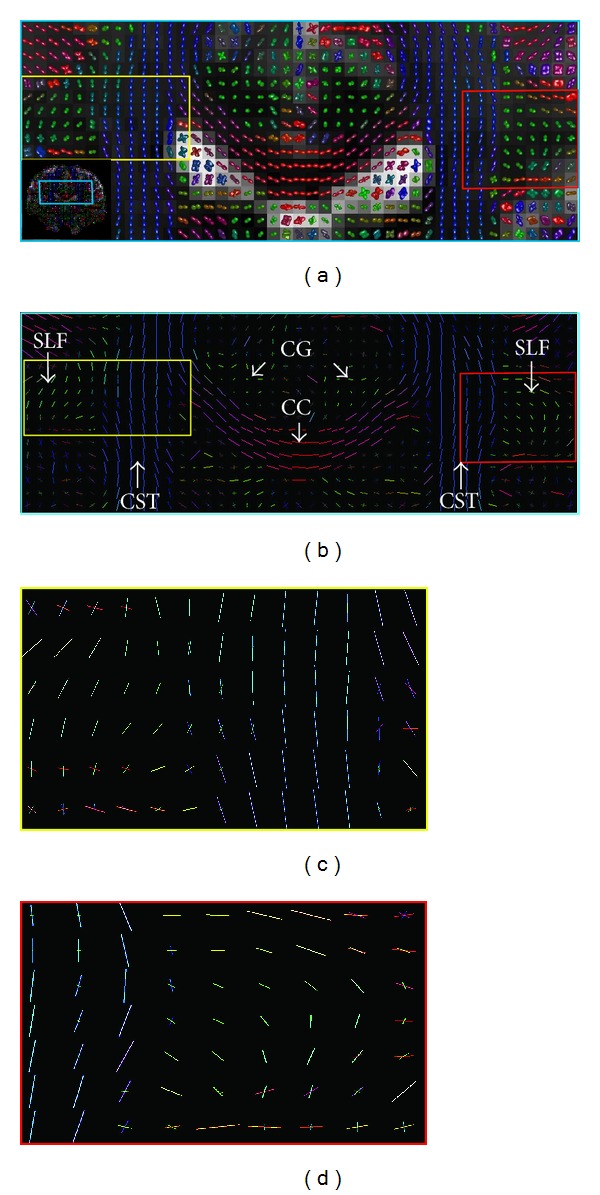
(a) Fourth order CT-FODs are reconstructed from* in vivo* human cerebral dataset and represent the Corpus Collum (CC), the Corticospinal Tract (CST), the Cingulum (CG), and the Superior Longitudinal Fasciculus (SLF) on a coronal slice; the CT-FODs are superposed on the raw diffusion signal. (b) represents the fiber directions (colored according to their directions) extracted using the Adecomp-SHOT based approach. Yellow and red highlighted areas are, respectively, zoomed in (c) and (d).

To validate our proposed crossing fibers detection method, we conduct many tests, first on synthetic diffusion dataset simulated with a multitensor model with 60 gradient directions and a *b*-value of 3000 s/mm^2^; these data represent crossing fibers with variable separating angles from 90° to 0°. On these synthetic diffusion data we have compared our method to other methods in literature such as CP-decomposition based method and the Z-eigenvalues based approach; the results of this comparison are illustrated in Figure 4. Then, the synthetic dataset is corrupted with a rician noise of different signal to noise ratio (SNR = 40, 30, 20, and 10); 100 trials of noise are performed for each SNR level and for each separating angle; the results of the effect of a rician noise are presented in Figures 5 and 6 and Table 1. Finally, our method is tested on phantom and on* in vivo* human brain diffusion data as illustrated in Figures 7 and 8.

### 3.1. Validations on Synthetic Diffusion Dataset

*Comparison with CP-Decomposition and Z-Eigenvalues Based Approaches*. In order to compare the angular resolution of our fiber extraction approach to the one of the CP-decomposition and the Z-eigenvalues based methods, we have conducted experiments on same noise-free synthetic diffusion dataset. From these data we reconstruct the fourth order CT-FODs and then we extract the maxima of the CT-FODs using the CP-decomposition, the Z-eigenvalues approach, and our proposed Adecomp-SHOT based approach. As it is required by the CP-decomposition, to decompose the fourth order CT-FOD we set the low-rank of the decomposition approximation to 2; that is, we assume that we know the number of fibers in the voxel. We recall that rather than the CP-decomposition our Adecomp-SHOT based approach does not require predefining the rank of the decomposition, that is, not require knowing a priori the number of fibers in the voxel. Moreover, to insure roughly the convergence of the ALS algorithm used by PARAFAC we initialize it by the eigenvectors of each mode of the fourth order tensor. This initialization is possible only for lower ranks up to 3, because the tensor is of dimension 3 and contains only 3 modes; for higher ranks, the initialization will be random which do not insure the convergence of the algorithm.

Figure 4 illustrates the results obtained by the CP-decomposition, the Z-eigenvectors, and the Adecomp-SHOT based approach. From Figure 4 we clearly notice that the tensor decomposition methods have a better angular resolution than the Z-eigenvalues classical method which is not able to recover crossing fibers orientations at angles below 60°. The results show too that if we assume that the number of fibers is a priori known, then both of CP-decomposition and Adecomp-SHOT based method are equivalent in terms of angular resolution and accuracy. Nevertheless, the CP-decomposition remains limited by the constraint of predefining the rank and by the convergence of the ALS algorithm. The proposed Adecomp-SHOT based approach efficiently solves these problems without loss of angular resolution or accuracy. With an order of tensors not exceeding four and without predefining the number of fibers in a voxel, the fiber orientations are recovered with an angular resolution limit of 30° and a mean error less than 4° up to separation angles of 36°.

*The Effect of a Rician Noise*. The most important aspect of the Adecomp-SHOT based method is its ability to render the number of fibers automatically without any assumption; therefore, we evaluate in 100 experiences on noisy synthetic data the success rate of our approach in detecting the number of crossing fiber bundles for different crossing angles. The synthetic data are corrupted with a Rician noise with different SNR levels of 40, 30, 20, and 10; the results are summarized in Table 1. Furthermore, in order to compare our results with the state-of-the-art, we conduct the same experiences for each of the CP-decomposition and the Z-eigenvalue approaches. The results in Table 1 show that up to a crossing angle of 48° in case of SNR 30 the success rate of our method is of 100%; this rate slightly decreases for a crossing angle of 42° where the correct number of fibers is rendered at 95%, and for a crossing angle of 36° the success rate is of 41%. Notice that, even in case of low signal to noise ratio SNR = 20, the correct number of fibers is found with a rate of 99% up to crossing angle of 48°, and for a really low SNR level of 10 the success rate is higher than 72% up to a separation angle of 54°. These results prove that the ability of the Adecomp-SHOT method to find automatically the number of fibers is not highly sensitive to noise. Thus, our Adecomp-SHOT based method is really reliable when we aim to detect the number of underlying fibers, even in case of really low SNR levels. For comparison, results about the number of fibers rendered by the CP and the Z-eigenvalues methods are too represented in Table 1; we can notice from these results that even if the number of fibers is automatically recovered by the Adecomp-SHOT approach, the success rate does not highly differ from the success rate of the CP-decomposition where the number of fibers constitutes an input parameter.

To evaluate the effect of noise on the accuracy of our Adecomp-SHOT based approach we represent on the left column of Figures 5 and 6 the mean and the standard deviation of the mean error between the recovered fiber orientations and the ground truth fiber orientations, corresponding to different SNR levels, and on the right column of Figures 5 and 6 we represent the mean and standard deviation of the fiber weights *λ*; figures on the center column of Figures 5 and 6 represent the mean and the standard deviation of the number of detected fibers. Once again, in order to compare our approach to other classical and state-of-the-art methods, results for each of our Adecomp-SHOT based approach CP and Z-eigenvalues approaches are illustrated on Figures 5 and 6. As in the free-noise case, Figures 4(a) and 4(b), for SNR = 40, Figures 5(a)–5(c), the method recovers effectively the entire simulated fiber orientations with an angular resolution limit equal to 30° and up to a separation angle of 36° the two crossing fibers are detected with a slight increase in the mean error but the mean of the mean error remains less than 9° as it is shown by Figure 5(a). While, for SNR 30, Figures 5(d)–5(f), the angular resolution limit is somewhat reduced and its value is between 36° and 30° with a mean of the mean error not exceeding 8° for a separating angle of 42° as shown in Figure 5(d); we notice that even when the SNR level equals 30, the angular resolution limit is still better than the angular resolution limit of the classical maxima localization methods in the free-noise case. For a low signal to noise ratio of 20, Figures 6(a)–6(c), the method still recovers the fiber orientations with an angular limit between 36° and 42° and the mean error remains inferior to 8° for a crossing angle of 48°. Up to SNR level of 20 the results of each of the Adecomp-SHOT and CP methods are equivalent in terms of angular resolution and accuracy if we assume the number of fiber known. Concerning the Z-eigenvalues approach, the results on Figure 5 confirm that even in case of a high SNR level the Z-eigenvalues method clearly fails to recovers crossing fiber orientations when the separation angle is lower than 60°. Furthermore, additional tests are performed with really low SNR level of 10 to evaluate much more the robustness to noise. The results are represented in Figures 6(d)–6(f) and show that in case of SNR 10 the angular resolution of our method is between 48° and 54° where the correct number of fibers is rendered at more than 67% (Table 1) with a mean of the mean error not exceeding 16° for a separation angle of 48° while for the same separation angle the CP-decomposition method has a mean of the mean error higher than 21°. These results prove that our proposed Adecomp-SHOT based approach is effective and robust to noise.

### 3.2. Phantom Diffusion Dataset

We conduct other experiments on the FiberCup phantom data acquired at *b* = 2000 s/mm^2^ with 64 acquisitions [21, 22] downloaded from the computer-assisted neuroimaging laboratory (LNAO) link: http://www.lnao.fr/spip.php?article112. The fourth order tensor is estimated from the phantom data using the CT-FOD algorithm and represented by spherical function in Figure 7(a). The recovered fiber directions are plotted on Figure 7(b). The results show that our Adecomp-SHOT based approach is able to render the fiber bundle directions.

### 3.3. *In Vivo* Human Cerebral Dataset

We conduct further tests, on real dataset obtained from the Stanford University [23] link: http://purl.stanford.edu/yx282xq2090. These data were acquired with 160 gradient directions with a *b*-value of 2000 s/mm^2^; the thickness of slice is 2 mm. The CT-FODs of fourth order are estimated from these data and decomposed with the Adecomp-SHOT; Figures 8(a) and 8(b) show the fourth order CT-FODs and the fiber directions, respectively. On a coronal slice, Figure 8 shows that the method reliably extracts the Corpus Collum (CC), the Corticospinal Tractu (CST), the Cingulum (CG), and the Superior Longitudinal Fasciculus (SLF). Two regions representing the CC, CST, and SLF intersections are highlighted on Figures 8(c) and 8(d) and show the ability of our approach to extract crossing fibers with a high angular resolution.

## 4. Conclusion

In this paper, we have proposed an Adecomp-SHOT based approach to extract the fiber directions from DW-MRI data. Till now, the CP-decomposition approach constitutes the state-of-the-art of the tensor decomposition applied to the fiber directions search in dMRI. Although the CP-decomposition method provides a better angular resolution and accuracy than the classical methods of maxima localization, this numerical method is suboptimal and suffers from two important inconveniences: the inability to ensure the convergence of the algorithm and the requirement to predefining the rank of the decomposition, that is, the number of fibers in the voxel. Thus, to overcome the considerable limits related to the CP-decomposition approach in the diffusion MRI, we propose a novel approach based on an analytical decomposition of symmetric high order tensor to extract the fiber directions in dMRI. Unlike CP-decomposition, our proposed approach is able to recover the entire crossing fiber directions whatever its number and that without any assumption. To exploit the Adecomp-SHOT on diffusion dataset and to take into account the ground truth properties of the diffusion, we imposed a real and nonnegative constraint by using the NNLS algorithm and by introducing a change of variables. The change of variables was done by a rotation transformation to preserve the fiber directions. This coordinate transformation permitted to overcom a significant constraint imposed by the original Adecomp-SHOT. Indeed, the Adecomp-SHOT, as initially described, enables to extract directions in the affine space whose first Cartesian coordinates are zero, but by using optimally the transformation coordinates, the entire fibers directions are recovered whatever its positions in the affine space. Different validations tests were conducted on synthetic noisy diffusion data, phantom, and real data. The tests on synthetic dataset have shown three principal advantages. (1) The Adecomp-SHOT based approach overcomes the limits related to CP-decomposition without loss in the angular resolution and angular accuracy. (2) Our approach is efficient, accurate, and robust to noise: for SNR equal to 30 our Adecomp-SHOT based approach has an angular resolution limit <36° and up to 42° the mean of the mean error does not exceed 8°. (3) The rician noise does not really affect the ability of the method to detect the number of fibers in a voxel; indeed, up to a crossing angle of 48° for a low SNR equal to 20 the correct number of fibers is found at 99%. Finally, the tests conducted on phantom and real data confirm the ability of the method to reliably extract the directions of fiber bundles especially in regions where many fiber bundles intersect.

## Figures and Tables

**Algorithm 1:**
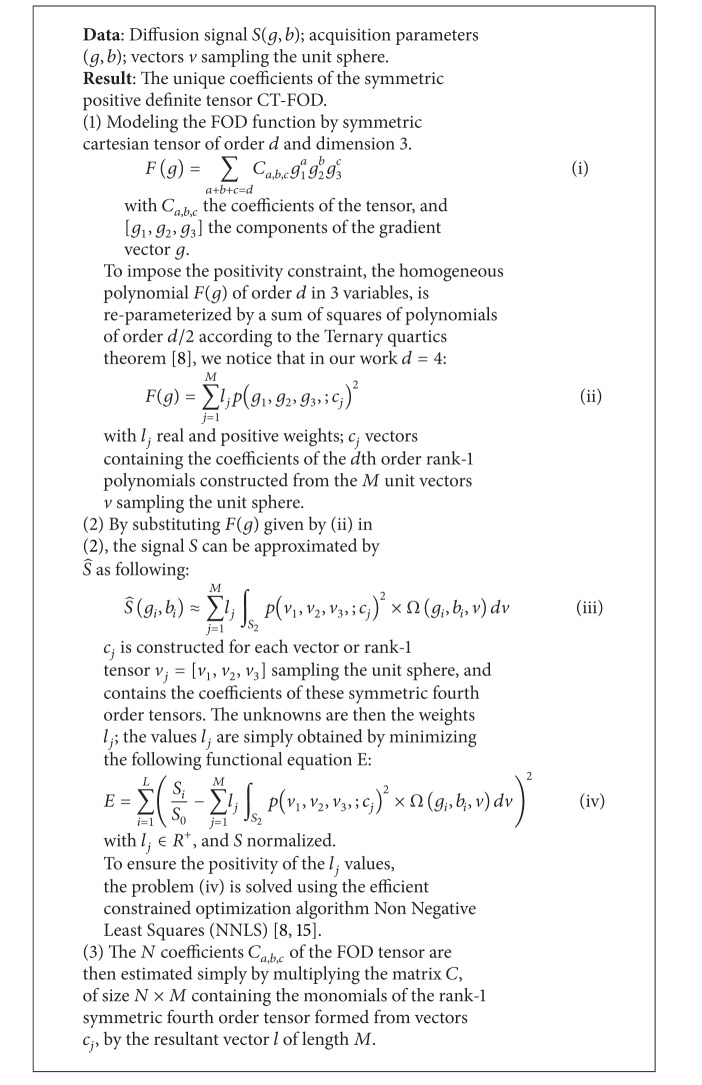


**Algorithm 2:**
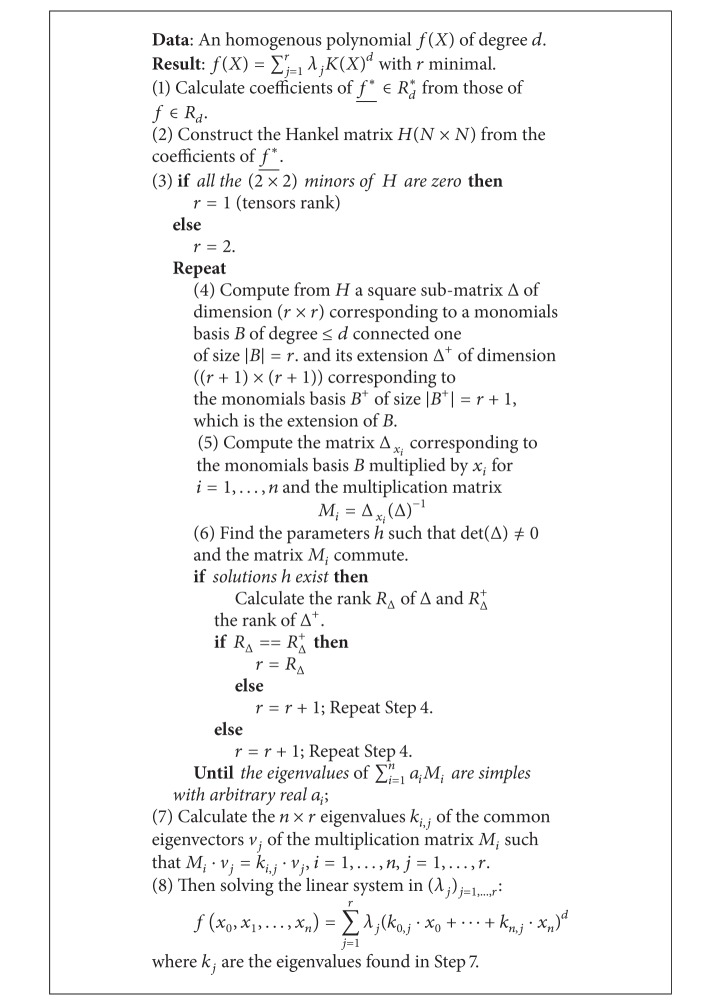


**Table 1:** Success rate (%) in detecting the number of crossing fiber bundles, results of 100 simulations of noise for each crossing angle and for each SNR level.

Separation angle		90°	84°	78°	72°	66°	60°	54°	48°	42°	36°	30°
SNR	Method											
40	Adecomp-SHOT	100%	100%	100%	100%	100%	100%	100%	100%	**100%**	68%	3%
	CP-decomp.	100%	100%	100%	100%	100%	100%	100%	100%	100%	61%	1%
	Z-eigenvalues	100%	100%	99%	99%	90%	11%	*0% *				

30	Adecomp-SHOT	100%	100%	100%	100%	100%	100%	100%	100%	**95%**	41%	4%
	CP-decomp	100%	100%	100%	100%	100%	100%	100%	100%	94%	39%	3%
	Z-eigenvalues	100%	100%	98%	100%	74%	11%	*0% *				

20	Adecomp-SHOT	99%	99%	99%	99%	98%	99%	98%	**99%**	77%	33%	8%
	CP-decomp	100%	100%	100%	100%	100%	100%	100%	99%	74%	34%	4%
	Z-eigenvalues	99%	100%	100%	99%	72%	24%	*0% *				

10	Adecomp-SHOT	96%	82%	88%	82%	80%	81%	**72%**	67%	54%	36%	27%
	CP-decomp	100%	100%	100%	100%	100%	100%	99%	92%	80%	67%	55%
	Z-eigenvalues	100%	99%	98%	95%	73%	47%	*0% *				

**Table 2:** The (11 × 11) part of the Hankel matrix constructed from the coefficients $\underline{C^{*}}$ of $\underline{f^{*}}$.

	1	*x*_1_	*x*_2_	*x*_1_^2^	*x*_1_ *x*_2_	*x*_2_^2^	*x*_1_^3^	*x*_1_^2^ *x*_2_	*x*_1_ *x*_2_^2^	*x*_2_^3^	*x*_1_^4^
1	0.145	0.0185	0.079	0.0938	− 0.0115	0.0588	0.0238	0.0149	0.127	0.0249	0.169
*x*_1_	0.0185	0.0938	− 0.0115	0.0238	0.0149	0.0127	0.169	− 0.0775	0.0538	− 0.0292	*h*_50
*x*_2_	0.079	− 0.0115	0.0588	0.0149	0.0127	0.169	− 0.0775	0.0538	− 0.0292	0.0274	*h*_41
*x*_1_^2^	0.0938	0.0238	0.0149	0.0169	− 0.0775	0.0538	*h*_50	*h*_41	*h*_32	*h*_23	*h*_60
*x*_1_ *x*_2_	− 0.0115	0.0149	0.0127	− 0.0775	0.0538	− 0.00292	*h*_41	*h*_32	*h*_23	*h*_14	*h*_51
*x*_2_^2^	0.0588	0.0127	0.0249	0.0538	− 0.0292	0.274	*h*_32	*h*_23	*h*_14	*h*_05	*h*_42
*x*_1_^3^	0.0238	0.169	− 0.0775	*h*_50	*h*_41	*h*_32	*h*_60	*h*_51	*h*_42	*h*_33	*h*_70
*x*_1_^2^ *x*_2_	0.0149	− 0.0775	0.0538	*h*_41	*h*_32	*h*_23	*h*_51	*h*_42	*h*_33	*h*_24	*h*_61
*x*_1_ *x*_2_^2^	0.0127	0.0538	− 0.0292	*h*_32	*h*_23	*h*_14	*h*_42	*h*_33	*h*_24	*h*_15	*h*_52
*x*_2_^3^	0.0249	− 0.0292	0.274	*h*_23	*h*_14	*h*_05	*h*_33	*h*_24	*h*_15	*h*_06	*h*_43
*x*_1_^4^	0.169	*h*_50	*h*_41	*h*_60	*h*_51	*h*_42	*h*_70	*h*_61	*h*_52	*h*_43	*h*_80

**Table 3:** Hankel matrix structure.

	*x*_1_^*b*_*m*_^ *x*_2_^*c*_*m*_^⋯
*x*_1_^*b*_*l*_^ *x*_2_^*c*_*l*_^	${\underline{C}}_{b_{l}+b_{m},c_{l}+c_{m}}^{*}\cdots$
⋮	⋮

## Appendix

As described by (7) of Section 2.2.2, the affine and minimal decomposition of a rank-4 polynomial *f* of degree 4 in 3 variables is given by the following form:

(A.1) $$\begin{array}{c} f\left(x_{0},x_{1},x_{2}\right)=\overset{4}{\underset{j=1}{\sum }}\lambda_{j}K_{j}{\left(x_{0},x_{1},x_{2}\right)}^{4},\\ K_{j}\left(x_{0},x_{1},x_{2}\right)=k_{j,0}\cdot x_{0}+k_{j,1}\cdot x_{1}+k_{j,2}\cdot x_{2}. \end{array}$$

According to (A.1), we generate a 4th order tensor of rank-4 in 3 dimensions from 4 crossing vectors *k*_*j*_ separated with angle of 63.43°; these vectors *k*_*j*_, given below, are uniformly distributed on the unit sphere and weighted with the same weights *λ*_*j*_ = 0.25. Consider

(A.2) $$\begin{array}{c} \left|\begin{array}{cccc} k_{1} & k_{2} & k_{3} & k_{4}\\ 0.00623 & -0.4 & 0.79 & 0.6367\\ 0.0644 & -0.828 & 0.385 & -0.6531\\ -0.998 & 0.392 & 0.478 & 0.41 \end{array}\right|. \end{array}$$

The resulted tensor is represented by the polynomial *f* given by

(A.3) f(x0,x1,x2)=0.145x04+0.0739x03x1+0.316x03x2+0.563x02x12−0.137x02x1x2+0.353x02x22+0.095x0x13+0.179x0x12x2+0.153x0x1x22+0.0998x0x23+0.169x14−0.31x13x2+0.323x12x22−0.117x1x23+0.274x24.

In the following, we propose to illustrate how the Adecomp-SHOT algorithm works by decomposing the homogenous polynomial *f* given in (A.3) step by step. *f* will be thus decomposed in a minimal sum of 4th order rank-1 terms as described in (A.1), and at the end we expect to find the vectors *k*_*j*_ given in the equation (A.2) and the weights *λ*_*j*_ = 0.25. An affine decomposition of *f* exists, if and only if an affine decomposition of $\underline{f^{*}}$ exists; decomposing *f* is then equivalent to decomposing $\underline{f^{*}}$, which is the unhomogenization of *f* in the dual space; then, the first step of the Adecomp-SHOT algorithm is the computation of the coefficients $\underline{C^{*}}$ of $\underline{f^{*}}$ from the coefficients *C* of *f*.

Step 1 . Consider computation of the coefficients $\underline{C^{*}}$ of $\underline{f^{*}}$ from the coefficients *C* of *f*. From the coefficients *C* of *f* we compute the coefficients *C** of *f** as described in (A.5), with *f** the associated linear form of *f* in the dual space *R*_4_*. Consider a general form of a 4th order tensor of dimension 3 given by

(A.4) $$\begin{array}{c} f\left(x_{0},x_{1},x_{2}\right)=\underset{a+b+c=4}{\sum }C_{a,b,c}x_{0}^{a}x_{1}^{b}x_{2}^{c}, \end{array}$$

(A.5) $$\begin{array}{c} C_{a,b,c}^{*}=\frac{a!b!c!}{4!}\times C_{a,b,c}. \end{array}$$

From the expression of *f* in (A.3) we deduce the coefficients *C* of *f* and by the relation (A.5) we calculate the coefficients *C** of *f** from the coefficients *C*:

(A.6) C=[0.145,0.0739,0.316,0.563,−0.137,0.353,0.095,0.179,0.153,0.0998,0.169,−0.31,0.323,−0.117,0.274],C∗=[0.145,0.0185,0.079,0.0938,−0.0115,0.0588,0.0238,0.0149,0.0127,0.0249,0.169,−0.0775,0.0538,−0.0292,0.274].

Then, the associated linear form *f** of *f* in the dual space *R*_4_* is given by the following expression:

(A.7) f∗=0.145x04+0.0185x03x1+0.079x03x2+0.0938x02x12−0.0115x02x1x2+0.0588x02x22+0.0238x0x13+0.0149x0x12x2+0.0127x0x1x22+0.0249x0x23+0.169x14−0.0775x13x2+0.0538x12x22−0.0292x1x23+0.274x24.

Computing $\underline{f^{*}}$ by unhomogenizing *f** according to *x*_0_,

(A.8) f∗_=0.145+0.0185x1+0.079x2+0.0938x12−0.0115x1x2+0.0588x22+0.0238x13+0.0149x12x2+0.0127x1x22+0.0249x23+0.169x14−0.0775x13x2+0.0538x12x22−0.0292x1x23+0.274x24.

The nonhomogenous basis of monomial is given by $\underline{B}$:

(A.9) B_=[1,x1,x2,x12,x1x2,x22,x13,x12x2,x1x22,x23,x14,x13x2,x12x22,x1x23,x24].

And the coefficients $\underline{C^{*}}$ of $\underline{f^{*}}$ are the same as the coefficients *C** of *f**:

(A.10) C∗_=[0.145,0.0185,0.079,0.0938,−0.0115,0.0588,0.0238,0.0149,0.0127,0.0249,0.169,−0.0775,0.0538,−0.0292,0.274].

Step 2 . Construct the Hankel matrix *H* of size (*N* × *N*) (Table 3) from $\underline{f^{*}}$; we notice that the elements corresponding to monomials with total degree higher than the polynomial degree 4 are unknown:

(A.11) H(l,m)=C_bl+bm,cl+cm∗ if  bl+bm+cl+cm≤4H(l,m)=hbl+bm,cl+cm(unknown)  else

with *l*, *m* = 1,…, *N*, the number of rows and columns, respectively, of the Hankel matrix *H*, and ${\underline{C}}_{b_{l}+b_{m},c_{l}+c_{m}}^{*}$corresponding to the monomial *x*_1_^*b*_*l*_+*b*_*m*_^ *x*_2_^*c*_*l*_+*c*_*m*_^. We denote by *N* the number of unique coefficients in the tensor; *N* is a function in the order *d* and dimension *n* and is calculated by

(A.12) $$\begin{array}{c} N=\frac{\left(n+\left(d-1\right)\right)!}{d!\left(n-1\right)!}. \end{array}$$

In our case *d* = 4 and *n* = 3, then *N* = 15.
Table 2 gives a (11 × 11) part of the Hankel matrix constructed from the coefficients $\underline{C^{*}}$ of $\underline{f^{*}}$. We give below an example on how to calculate the Hankel matrix elements from the coefficients $\underline{C^{*}}$ of the polynomial $\underline{f^{*}}$ given by (A.8):

H(1,1)=C_0,0∗ corresponding to the monomial *x*_1_^0^ *x*_2_^0^;
*H*(1,1) = 0.145;
$H(2,4)={\underline{C}}_{3,0}^{*}$ corresponding to the monomial *x*_1_^3^ *x*_2_^0^;
*H*(2,4) = 0.0238;
$H(5,7)={\underline{C}}_{4,1}^{*}$ corresponding to the monomial *x*_1_^4^ *x*_2_^1^;
*H*(5,7) = *h*_41_ (unknown);
$H(10,8)={\underline{C}}_{5,1}^{*}$ corresponding to the monomial *x*_1_^5^ *x*_2_^1^;
*H*(10,8) = *h*_51_ (unknown).

Step 3 . Check if the polynomial *f* is of rank-1. For that, we check if all the minors (2 × 2) that we can calculate from *H* are equal to zero. In our case, all the minors (2 × 2) of *H* are not zero; then we set *r* = 2.

Step 4 . Find the rank of the tensor iteratively. For *r* = 2, we compute from *H* a square submatrix Δ of dimension (*r* × *r*) corresponding to a monomials basis *B* = [1, *x*_1_] connected to one of size |*B* | = *r*. Consider

(A.13) Δ=|1x10.1450.01850.01850.0938|1x1

and its extension Δ^+^ of dimension ((*r* + 1)×(*r* + 1)) corresponding to the monomials basis *B* ^+^ = [1, *x*_1_, *x*_2_] of size |*B* ^+^ | = *r* + 1, which is the extension of *B*. Consider

(A.14) $$\begin{array}{c} \Delta^{+}=\left|\begin{array}{ccc} 1 & x_{1} & x_{2}\\ 0.145 & 0.0185 & 0.079\\ 0.0185 & 0.0938 & -0.015\\ 0.079 & -0.0115 & 0.0588 \end{array}\right|\begin{array}{c} 1\\ x_{1}\\ x_{2} \end{array}. \end{array}$$

Since Δ and Δ^+^ are totally defined, we just have to find the ranks *R* and *R* ^+^ of each of them and verify if the rank remains stable.
*R* = rank(Δ) = 2; *R* ^+^ = rank(Δ^+^) = 3,  *R* ≠ *R* ^+^; the stability condition of the rank is not satisfied; then, we increment *r* = *r* + 1. Repeat for *r* = 3

(A.15) $$\begin{array}{c} \Delta =\left|\begin{array}{ccc} 1 & x_{1} & x_{2}\\ 0.145 & 0.0185 & 0.079\\ 0.0185 & 0.0938 & -0.015\\ 0.079 & -0.0115 & 0.0588 \end{array}\right|\begin{array}{c} 1\\ x_{1}\\ x_{2} \end{array}\\ \Delta^{+}=\left|\begin{array}{cccc} 1 & x_{1} & x_{2} & x_{1}^{2}\\ 0.145 & 0.0185 & 0.079 & 0.0938\\ 0.0185 & 0.0938 & -0.015 & 0.238\\ 0.079 & -0.0115 & 0.0588 & 0.0149\\ 0.0938 & 0.0238 & 0.0149 & 0.169 \end{array}\right|\begin{array}{c} 1\\ x_{1}\\ x_{2}\\ x_{1}^{2} \end{array}. \end{array}$$

*R* = rank(Δ) = 3; *R* ^+^ = rank(Δ^+^) = 4,  *R* ≠ *R* ^+^; the stability condition of the rank is not satisfied; then, we increment *r* = *r* + 1. Repeat for *r* = 4

(A.16) $$\begin{array}{c} \Delta =\left|\begin{array}{cccc} 1 & x_{1} & x_{2} & x_{1}^{2}\\ 0.145 & 0.0185 & 0.079 & 0.0938\\ 0.0185 & 0.0938 & -0.015 & 0.238\\ 0.079 & -0.0115 & 0.0588 & 0.0149\\ 0.0938 & 0.0238 & 0.0149 & 0.169 \end{array}\right|\begin{array}{c} 1\\ x_{1}\\ x_{2}\\ x_{1}^{2} \end{array} \end{array}$$

(A.17) Δ+ =|1x1x2x12x1x20.1450.01850.0790.0938−0.0150.01850.0938−0.0150.2380.01490.079−0.01150.05880.01490.01270.09380.02380.01490.169−0.0775−0.01150.01490.0127−0.07750.0558|1x1x2x12x1x2.

*R* = rank(Δ) = 4; *R** = rank(Δ) = 4, *R* = *R* ^+^; the stability condition of the rank or equivalently the commutation properties of the multiplication matrix are satisfied.

Step 5 . Compute the matrices Δ_*x*_1__ and Δ_*x*_2__ corresponding to the monomials basis *B*_*x*_1__ = [*x*_1_, *x*_1_^2^, *x*_1_ *x*_2_, *x*_1_^3^] and *B*_*x*_2__ = [*x*_2_, *x*_1_ *x*_2_, *x*_2_^2^, *x*_1_^2^ *x*_2_], respectively, and the associated multiplication matrix *M*_*x*_1__ and *M*_*x*_2__, for *r* = 4. The bases *B*_*x*_1__ and *B*_*x*_2__ correspond to the monomial basis *B* = [1, *x*_1_, *x*_2_, *x*_1_^2^] multiplied by *x*_1_ and *x*_2_, respectively:

(A.18) Δ=|1x1x2x120.1450.01850.0790.09380.01850.0938−0.0150.2380.079−0.01150.05880.01490.09380.02380.01490.169|1x1x2x12Δx1=|x1x12x1x2x130.01850.0938−0.0150.2380.09380.02380.01490.0169−0.01150.01490.0127−0.07750.02380.169−0.0775h_50|1x1x2x12Δx2=|x2x1x2x22x12x20.079−0.01150.05880.0149−0.01150.01490.0127−0.0775−0.05880.0127−0.02490.05380.0149−0.07750.0588h_41|1x1x2x12

Constructing the multiplication matrix,

(A.19) Mxi=Δxi(Δ)−1M1=|01.0000001.0−9.512.7412.73.328299−4.14×104×h_501.04×10.4×h_50−20885.34×104×h_50−1.07×1041.69×10.4×h_50−3377|M2=|0001.0−9.512.7412.73.32159.0−39.9−205.0−64.4−4.14×10.4×h_41−3577.01.04×10.4×h_41+897.05.34×10.4×h_41+4600.01.69×10.4×h_41+1455.0|.

Step 6 . Find the parameters *h* such that det⁡(Δ) ≠ 0 and the matrix *M*_*x*_*i*__ commute. The commutation properties *M*_*x*_*i*__ *M*_*x*_*j*__ − *M*_*x*_*i*__ *M*_*x*_*j*__ = *∅* lead to 16 nonlinear equations, with only 5 nontrivial equations to solve; a solution of this problem is [*h*_41, *h*_50] = [−0.0835,0.2]. Now, we verify if the rank *r* = 4 is effectively the rank of the tensor by verifying the last condition, which is the multiplicity of the eigenvalues of ∑_*i*=1_^*n*^ *a*_*i*_ *M*_*i*_. Thus, if *L* = eig(∑_*i*=1_^*n*^ *a*_*i*_ *M*_*i*_) are simple with a random real value of *a*_*i*_, then *r* is effectively the rank of the tensor.
*L* = [0.8756,0.4123, −0.3946, −33.9557]; the eigenvalues *L* are simple; then the rank of the tensor is *r* = 4.

Step 7 . Solve the generalized eigenvalues problem for rank *r* = 4: calculate the *n* × *r* eigenvalues *k*_*i*,*j*_ of the common eigenvectors *v*_*i*_(:, *j*) of the multiplication matrix *M*_*x*_*i*__, with *i* = 0,1, 2 and *j* = 1,2, 3,4:

(A.20) $$\begin{array}{c} v_{1}=\left|\begin{array}{cccc} -0.0052 & 0.2014 & 0.7760 & 0.5389\\ -0.0536 & 0.4171 & 0.3783 & -0.5426\\ 0.8302 & -0.1974 & 0.4697 & 0.3406\\ -0.5549 & 0.8640 & 0.1844 & 0.5566 \end{array}\right|\\ v_{1}\left(:,1\right)=\left|\begin{array}{c} -0.0052\\ -0.0536\\ 0.8302\\ -0.5549 \end{array}\right|\\ l_{1}=\left|\begin{array}{cccc} 10.3513 & 0 & 0 & 0\\ 0 & 2.0715 & 0 & 0\\ 0 & 0 & 0.4875 & 0\\ 0 & 0 & 0 & -1.0258 \end{array}\right|\\ l_{1}=10.3513. \end{array}$$

*v*_1_ and *l*_1_ are, respectively, the eigenvectors and the eigenvalues of *M*_*x*_1__. Consider

(A.21) $$\begin{array}{c} v_{2}=\left|\begin{array}{cccc} -0.0052 & 0.2014 & 0.7760 & 0.5389\\ -0.0536 & 0.4171 & 0.3783 & -0.5426\\ 0.8302 & -0.1974 & 0.4697 & 0.3406\\ -0.5549 & 0.8640 & 0.1844 & 0.5566 \end{array}\right|\\ v_{2}\left(:,1\right)=\left|\begin{array}{c} -0.0052\\ -0.0536\\ 0.8302\\ -0.5549 \end{array}\right|\\ l_{2}=\left|\begin{array}{cccc} -160.2909 & 0 & 0 & 0\\ 0 & -0.9806 & 0 & 0\\ 0 & 0 & 0.6053 & 0\\ 0 & 0 & 0 & 0.6439 \end{array}\right|\\ l_{2}\left(1,1\right)=-160.2909. \end{array}$$

*v*_2_ and *l*_2_ are, respectively, the eigenvectors and the eigenvalues of *M*_*x*_2__. We construct the elements of the vectors *k*_*j*_ from the eigenvalues *l*_*i*_ of the common eigenvectors *v*_*i*_(:, *j*), with the first element corresponding to the unhomogenization variable *x*_0_ equal to 1 as follows. If *v*_1_(:, *j*) = *v*_2_(:, *j*), then

(A.22) $$\begin{array}{c} k_{j}=\left|\begin{array}{c} 1\\ l_{1}\left(j,j\right)\\ l_{2}\left(j^{\prime },j^{\prime }\right) \end{array}\right|\;\text{with}\;j,j^{\prime }=1,\ldots ,r. \end{array}$$

For instance, *v*_1_(:, 1) = *v*_2_(:, 1); then

(A.23) k1=|1l1(1,1)l2(1,1)| with  j,j′=1,…,r|k1k2k3k4111110.13132.07150.4875−1.0258−160.2909−0.95060.60530.6439|x0x1x2.

By normalizing the vectors *k*_*j*_, we get

(A.24) $$\begin{array}{c} \left|\begin{array}{cccc} k_{1} & k_{2} & k_{3} & k_{4}\\ 0.0062 & 0.3999 & 0.7896 & 0.6367\\ 0.0644 & 0.8284 & 0.3849 & -0.6531\\ -0.9979 & -0.3922 & 0.4779 & 0.4099 \end{array}\right|. \end{array}$$

The resulted *k*_*j*_ corresponds exactly to the original vectors used to construct the tensor.

Step 8 . Then we resolve the linear system in (*λ*_*j*_)_*j*=1,…,4_:

(A.25) f(x0,x1,x2) =∑j=14λj(k0,j·x0+k1,j·x1+k2,j·x2)4.

The solution of the above system is the following:

(A.26) $$\begin{array}{c} \lambda =\left[0.25,0.25,0.25,0.25\right]. \end{array}$$

Thus, the minimal decomposition of the symmetric fourth order tensor of rank-4 in 3 dimensions associated with the homogeneous polynomial of degree 4 in 3 variables *f*(*x*_0_, *x*_1_, *x*_2_) is given as follows:

(A.27) f(x0,x1,x2) =0.25(0.0062x0+0.0644x1−0.9979x2)4  +0.25(0.6367x0−0.6531x1+0.4099x2)4  +0.25(0.3999x0+0.8284x1−0.3922x2)4  +0.25(0.7896x0+0.3849x1+0.4779x2)4.

## Abbreviations

MRI: Magnetic resonance imaging
dMRI: Diffusion magnetic resonance imaging
DTI: Diffusion tensor imaging
HARDI: High angular resolution imaging
DSI: Diffusion spectrum imaging
QBI: Q-ball imaging
SHOT: Symmetric high order tensor
ODF: Diffusion orientation distribution function
FOD: Fiber orientation distribution function
SH: Spherical harmonics
CT-FOD: Cartesian tensor-FOD
CP: CANDECOMP/PARAFAC
ALS: Alternating least squares
Adecomp-SHOT: Analytical decomposition of a symmetric high order tensor
NNLS: Nonnegative least squares
SNR: Signal to noise ratio
CC: Corpus Callosum
CST: Corticospinal Tract
CG: Cingulum
SLF: Superior Longitudinal Fasciculus.
